# Variable structure diversification by multicatalysis: the case of alcohols

**DOI:** 10.1039/d3cc00551h

**Published:** 2023-03-20

**Authors:** Bruno Lainer, Kuhali Das, Paweł Dydio

**Affiliations:** a University of Strasbourg, CNRS, ISIS UMR 7006, 8 allée Gaspard Monge 67000 Strasbourg France dydio@unistra.fr

## Abstract

Given that alcohol moieties are present in a great diversity of valuable fine chemicals from nature and synthesis, methods enabling their structure diversification are highly sought after. Catalysis proved to enable the development of new transformations that are beyond the inherent reactivity of alcohols. However, modifying the structure of alcohols at certain unbiased positions remains a major challenge or requires tedious multistep procedures. Recently, increased attention has been given to multicatalyis, which combines multiple reactions and catalysts within one system, creating room for discovering previously inaccessible reactivities or increasing the overall efficiency of multistep transformations. This feature article focuses on demonstrating various aspects of devising such multicatalytic systems that modify the structure of alcohol-containing compounds. Special attention is given to highlighting the challenges and advantages of multicatalysis, and in a broader context discussing how the field of catalysis may progress toward more complex systems.

## Abstract 2

Given that alcohol moieties are present in a great diversity of valuable fine chemicals from nature and synthesis, methods enabling their structure diversification are highly sought after.

## Graphical abstract

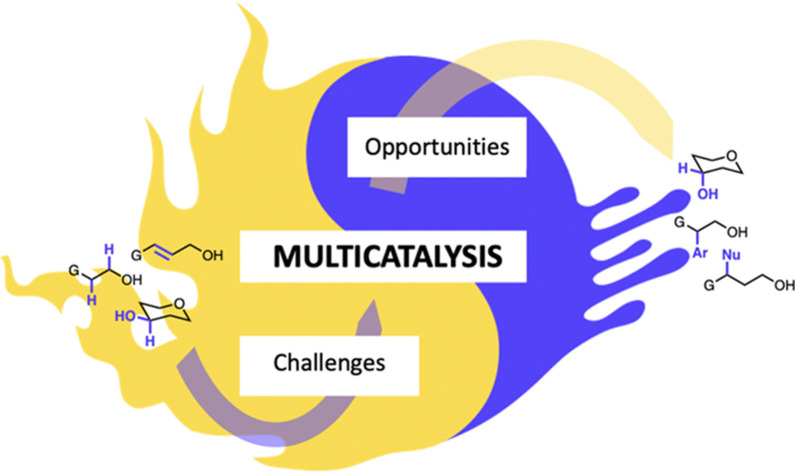

## Introduction

Alcohols are ubiquitous functional groups in natural products, synthetic bioactive compounds, pharmaceuticals and agrochemicals, commercial building blocks, and many other fine and bulk chemicals.^1–4^ Hence, methods enabling facile diversification of available alcohols toward libraries of structurally varied derivatives are highly sought after. Such strategies create opportunities to readily explore broad chemical space, facilitate the discovery and development of biologically relevant molecules, synthesize known valuable fine chemicals, and valorise feedstock materials.

The reactivity of alcohols is typically dictated by the lone pairs of the oxygen atom. Therefore, they often act as nucleophiles, or alternatively, as a proton source, due to the strong polarization of the O–H bond.^5^ In some instances, the hydroxyl group may serve as a directing group or can be easily transformed into convenient leaving groups.^6,7^ These modes of reactivity created room for the development of many useful transformations of alcohols. However, modifying alcohols at one of the neighbouring carbon positions remains cumbersome and typically requires wasteful multistep procedures. Therefore, alternative new strategies that overcome their limited inherent reactivity and enable their direct functionalization would be attractive.

Catalysis creates an opportunity to devise novel, valuable reactions.^8,9^ Carefully designed catalysts can execute transformations that modify molecules with high regio-, stereo-, and enantiocontrol under mild conditions. However, these reactions typically occur at inherently reactive and sterically accessible sites and altering compounds at sterically or electronically unbiased positions is still one of the major challenges in catalysis.^10^ Usually, such transformations require changing the reactivity of the molecule by introducing either new handles, such as catalyst-directing groups, or modifying the functional groups, conducted at the expense of additional tedious synthetic steps.^11^

Recently, inspired by biological systems, a trend has emerged in the field of catalysis, which involves using multiple catalysts to execute several transformations in one vessel, increasing the general efficiency and creating new selective transformations.^12–22^ For instance, multicatalysis proved to enable modifying molecules at their inherently poorly reactive sites thanks to the cooperativity between two orthogonal reactions operating in a one-pot. Also, merging multiple reactions into a one-step process makes the overall transformations more time-efficient, economical, and easier to execute for an experimenter. In general, the multicatalytic approaches have the potential to address many challenges of modern organic synthesis, unlocking new synthetic routes and novel derivatizations of molecules.

A historical example of multicatalysis outperforming traditional catalysis can be traced to the coupling of aryl halides with acetylenes. The groups of Cassar and Heck demonstrated that this reaction was possible using palladium catalysts.^23,24^ However, in a simultaneous report, the group of Sonogashira demonstrated that adding copper salt as a co-catalyst allows for conducting the reaction at milder conditions.^25^ Overall, the presence of two catalysts was crucial for expanding the application of the transformation.^26^

**Fig. 1:**
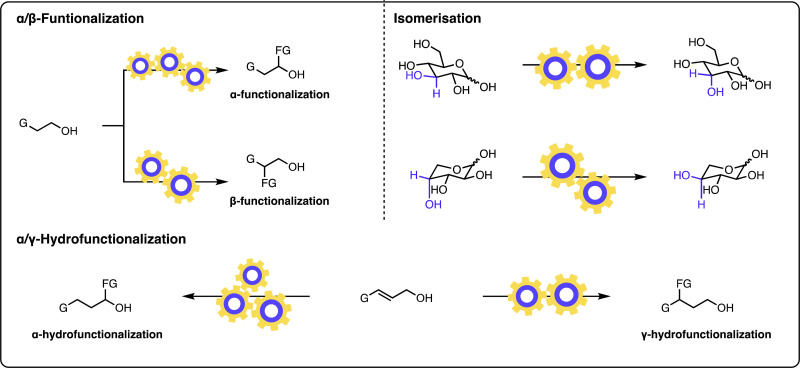
Multicatalytic approaches to modifying alcohols: α-functionalization of alcohols, β-functionalization of alcohols, α-hydro functionalization of allylic alcohols, γ-hydro functionalization of allylic alcohols, site-selective modifications of sugars.

The field of multicatalysis has undergone vigorous growth over recent years, enabling a broad range of highly selective transformations for various classes of starting materials, and has been the subject of multiple reviews.^12–22^ In this Feature article, we focus on different aspects of the design and development of such multicatalytic systems, their advantages, and their challenges, for modifying the structure of alcohol-containing molecules (Fig. 1). More specifically, the focus will be paid to transformations of alcohols as starting materials in which the hydroxyl group remains unaltered in the product. Multicatalytic approaches, which convert the alcohol group into a different functionality, are outside of the scope of this feature article.^14,27–30^

### α-Functionalization of aliphatic alcohols

**Scheme 1:**
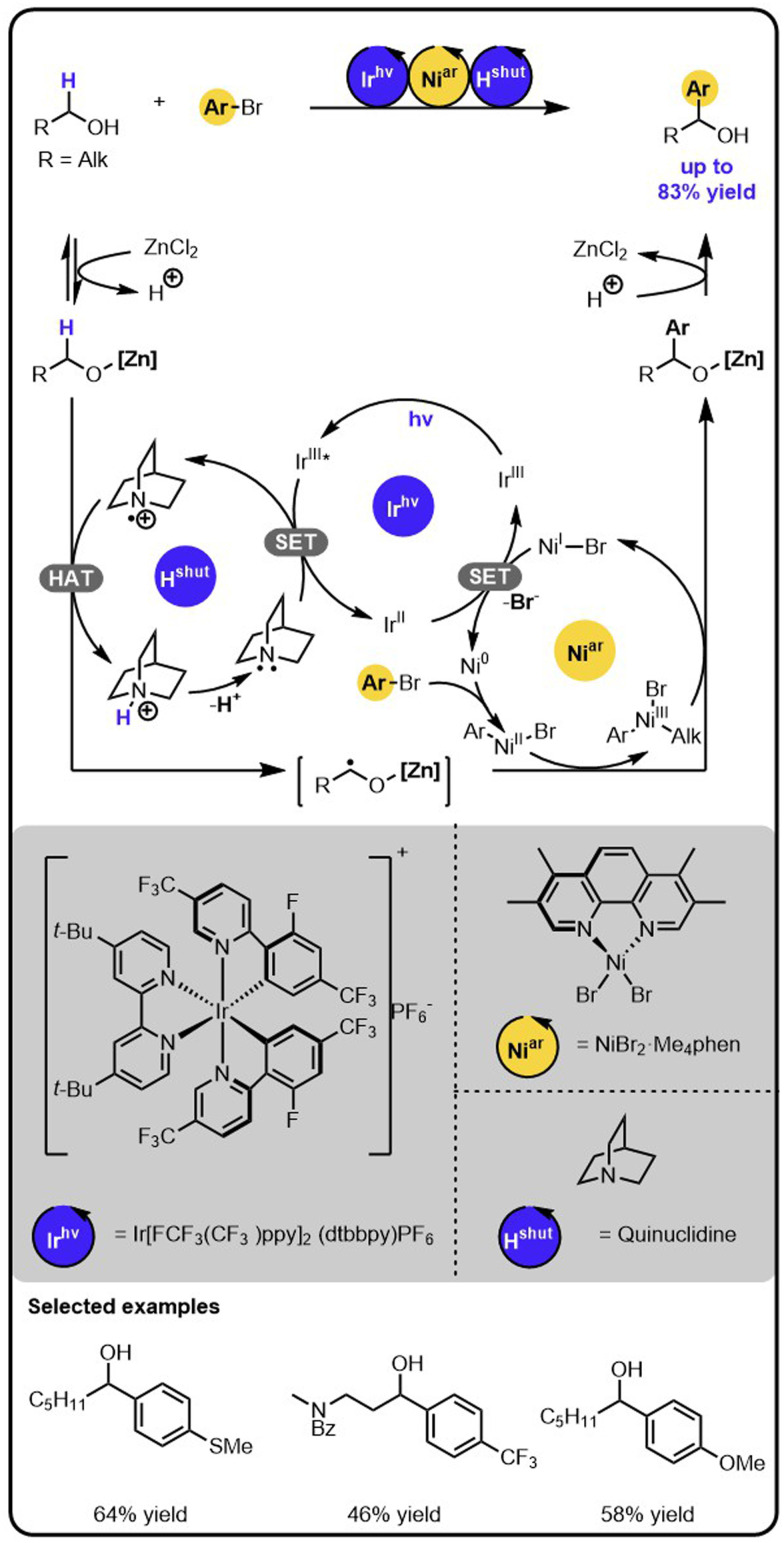
α-Arylation of alcohols with aryl bromides under the Ir/Ni/amine photoredox multicatalysis.

MacMillan and coworkers showed that the merger of nickel catalysis with photoredox catalysis enables the direct α-arylation of alcohols with aryl bromides (Scheme 1).^31^ The identification of the conditions required the evaluation of various nickel catalysts, iridium photocatalysts, bases, and Lewis acids that promoted the productive pathway and suppressed side-processes. Specifically, first, the aminium radical cation, H^shut^, needs to be generated by the oxidation of an amine with the excited iridium photocatalyst in a single electron transfer (SET). Next, the hydrogen atom transfer (HAT) occurs between H^shut^ and the alkoxide to generate an α-carbon-centered radical. The latter then adds to a nickel(ii) aryl halide complex, which is generated before through the oxidative addition of aryl bromide to a nickel (0) complex. The resulting nickel(iii) alkyl aryl intermediate undergoes reductive elimination, forming the product and a nickel(i) species. Lastly, the nickel(i) intermediate is reduced back to nickel (0) complex in a SET event oxidizing the initially reduced iridium photocatalyst, thereby regenerating all intermediates involved and closing all catalytic cycles.

The critical aspects to enabling the cooperation of the different catalysts were suppressing the competitive C–O arylation and the chemoselective formation of a radical in the α-position of the alcohols. Crucial, as it turned out, was using an appropriate Lewis acid to activate the desired position by forming a metal alkoxide. The α-C–H bond of this species has an increased hydridic character that promotes the formation of the α-carbon-centred radical by hydrogen abstraction. Also, the Lewis acid is key to preventing transmetalation of the alkoxide species with nickel(ii) aryl intermediates, the side process that could be followed by reductive elimination leading to the formation of aryl ethers.^32–34^

Accessing secondary benzylic alcohols *via* a dual-catalytic photoredox system is also possible, starting from α-hydroxyalkyltrifluoroborates as the radical precursor, as was demonstrated by the group of Molander.^35^ Along the same lines, the α-alkylation of alcohols has recently been reported by Kanai and coworkers.^36^ Here, a merger of a photocatalyst, a hydrogen atom transfer catalyst, and a Lewis acid catalyst mediates generating α-carbon-centred radical, which adds to an alkene to yield secondary alcohol.

**Scheme 2:**
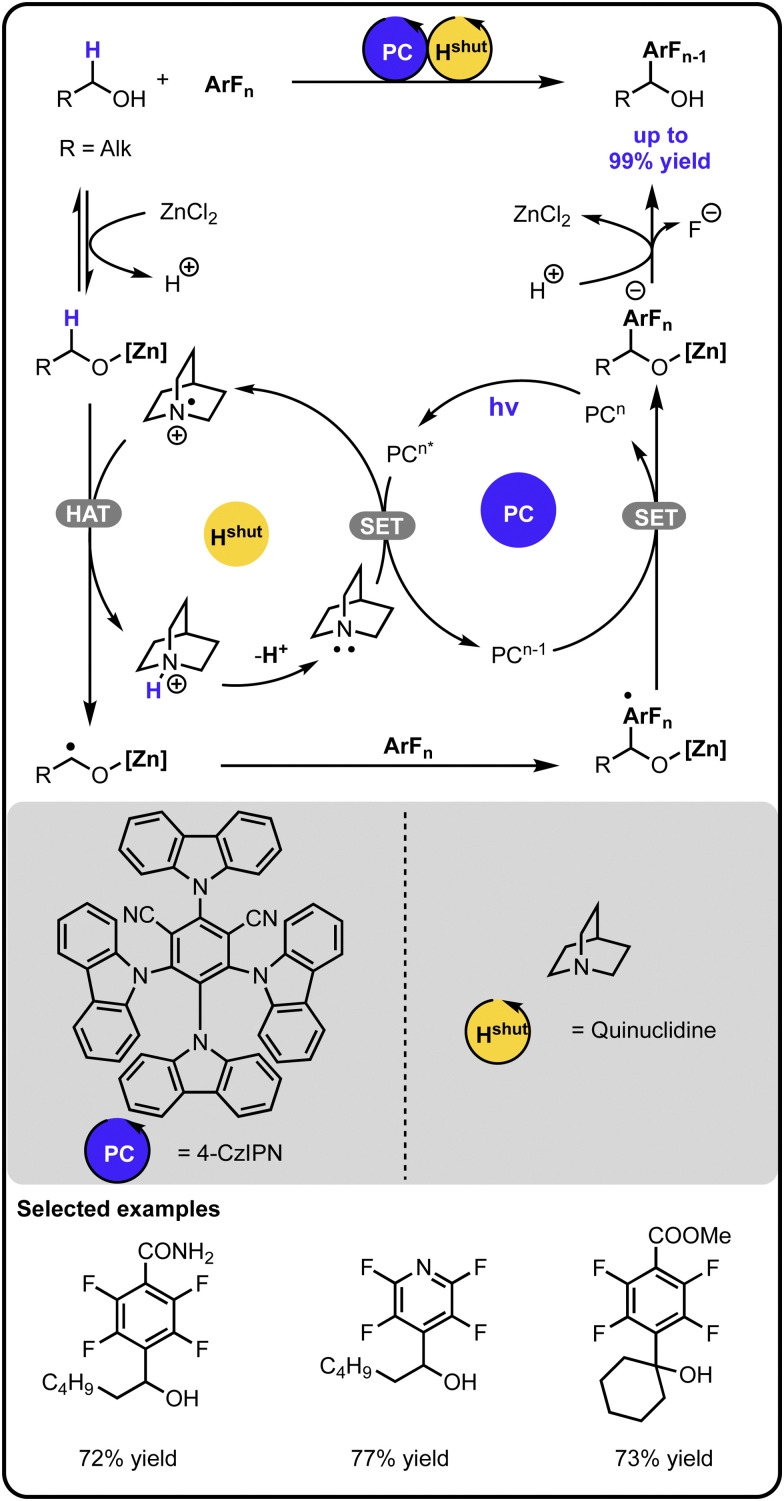
α-Arylation of alcohols with aryl fluorides under the photoredox-hydrogen atom transfer multicatalysis.

In 2022, Wu and coworkers expanded the chemistry of radical-mediated α-arylation of alcohols with polyfluoroarenes *via* a cooperative photoredox-hydrogen atom transfer catalysis (Scheme 2).^37^ A Lewis acid was involved in the initial activation of the alcohol, which upon hydrogen atom abstraction by a quinuclidine radical cation, generates an α-carbon-centered radical. The latter adds to the aryl fluoride, and a subsequent single electron transfer followed by fluoride elimination and proton exchange leads to the product.

The same type of transformation, *i.e.*, α-arylation of alcohols, can also be carried out in non-catalytic systems; however, their synthetic utility is somewhat limited when compared to the multicatalytic approach.^38,39^ Having catalysts controlling each step of the transformations allows for higher selectivity compared to non-catalytic approaches, permitting higher functional group tolerance. For instance, the non-catalytic methods appear to not operate well with electron-rich aryl halides or tolerate functional groups such as amines or sulphides. Additionally, this type of transformation is also feasible in a system with only one catalyst present, as demonstrated by the nickel catalysed α-arylation of alcohols reported by the group of Newman.^40,41^

**Scheme 3:**
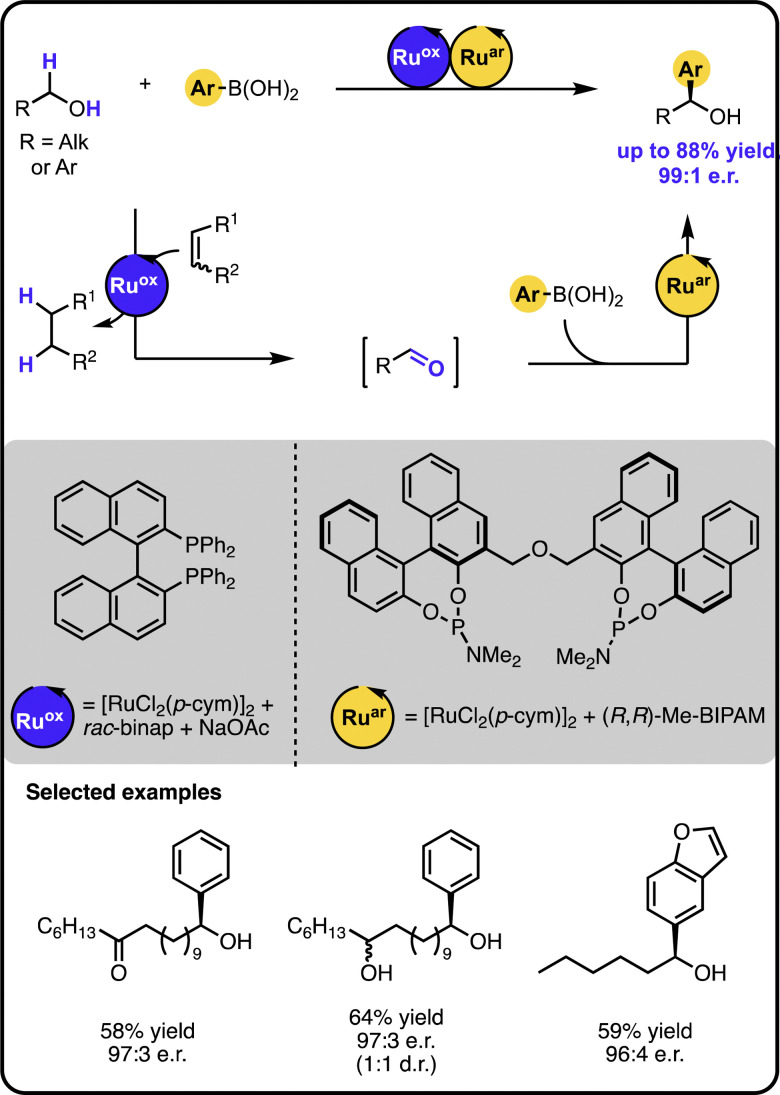
Enantioselective α-arylation of alcohols with boronic acids under the Ru/Ru sequential dual-catalytic relay.

Our group reported enantioselective α-arylation of primary aliphatic alcohols under multicatalysis furnishing secondary benzylic alcohols in high yields and enantioselectivity (Scheme 3).^42^ The method rested on a sequential relay system combining an oxidation step with a subsequent hydroarylation step, both of which were executed by suitable Ru catalysts. A sequential relay enabled overcoming incompatibilities between the two catalysts and allowing for adjusting reaction conditions for both steps. Initially, the alcohol is oxidized to the aldehyde by a ruthenium-catalysed hydrogen transfer to a sacrificial acceptor. The oxidation catalyst used preferentially targets primary alcohols over secondary ones, creating a possibility to perform a site-selective functionalization of a molecule containing diverse alcohol moieties. Upon oxidation, a ruthenium-hydroarylation catalyst performs the enantioselective 1,2-addition of boronic acids to the aldehyde intermediate, yielding the secondary benzyl alcohol product. The 1,2-addition step was selective towards an aldehyde moiety, enabling the functionalisation of alcohols bearing other carbonyl-containing functional groups, such as ketones, esters, or amides.

In developing the above-described dual catalyst protocol, it was pivotal to find an appropriate combination of a hydrogen transfer catalyst and hydrogen acceptors that would not interfere with the catalyst for the 1,2-addition cycle. For instance, the hydrogen transfer catalyst should not be active in the addition reaction and its ligand should not exchange with the ligand of the addition catalyst, since ligand scrambling often causes deterioration in the observed activity and enantioselectivity.^22^ The selected hydrogen transfer catalyst had no adverse effect on the hydroarylation catalyst, and the hydrogen acceptor used did not impact the addition step of the sequential relay.

An example with similar working principles is the α-borylation of alcohols. Here a ruthenium-based catalyst oxidizes the alcohol to an aldehyde using a sacrificial hydrogen acceptor.^43^ Next, an iron species allows for the borylation of the carbonyl intermediate to yield the product. This work demonstrates the potential of multicatalysis to introduce heteroatoms into alcohol's framework.

**Scheme 4:**
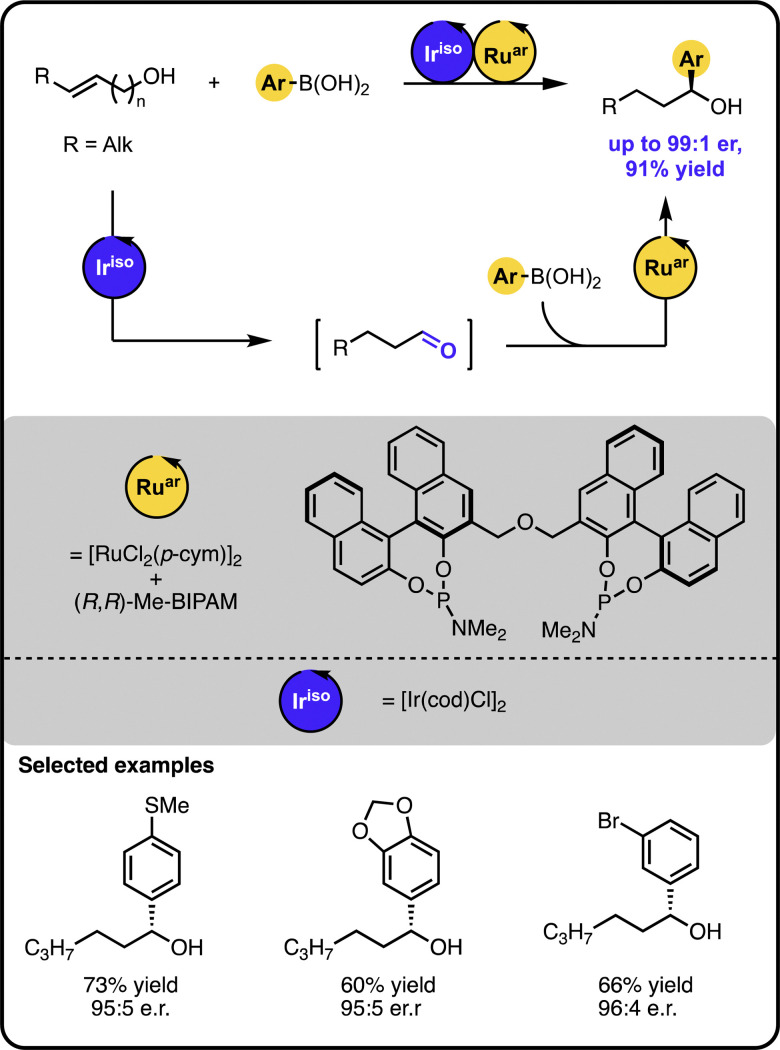
Enantioselective synthesis of secondary benzylic alcohols starting from unsaturated alcohols and boronic acids under the Ir/Ru sequential dual-catalytic relay.

Our group also demonstrated that enantioenriched secondary benzylic alcohols could be obtained in a one-pot process starting from (homo)allylic alcohols and aryl boronic acids (Scheme 4).^44^ Here, an initial iridium-catalysed isomerization of the starting alcohol forms an aldehyde intermediate. Subsequently, a ruthenium-based catalyst facilitates the enantioselective 1,2-additon of an aryl boronic acid to the aldehyde intermediate. It was also possible to functionalize alcohols bearing more remote double bonds, albeit the process required a ruthenium “Zipper” catalyst to enable effective isomerisation.

**Scheme 5:**
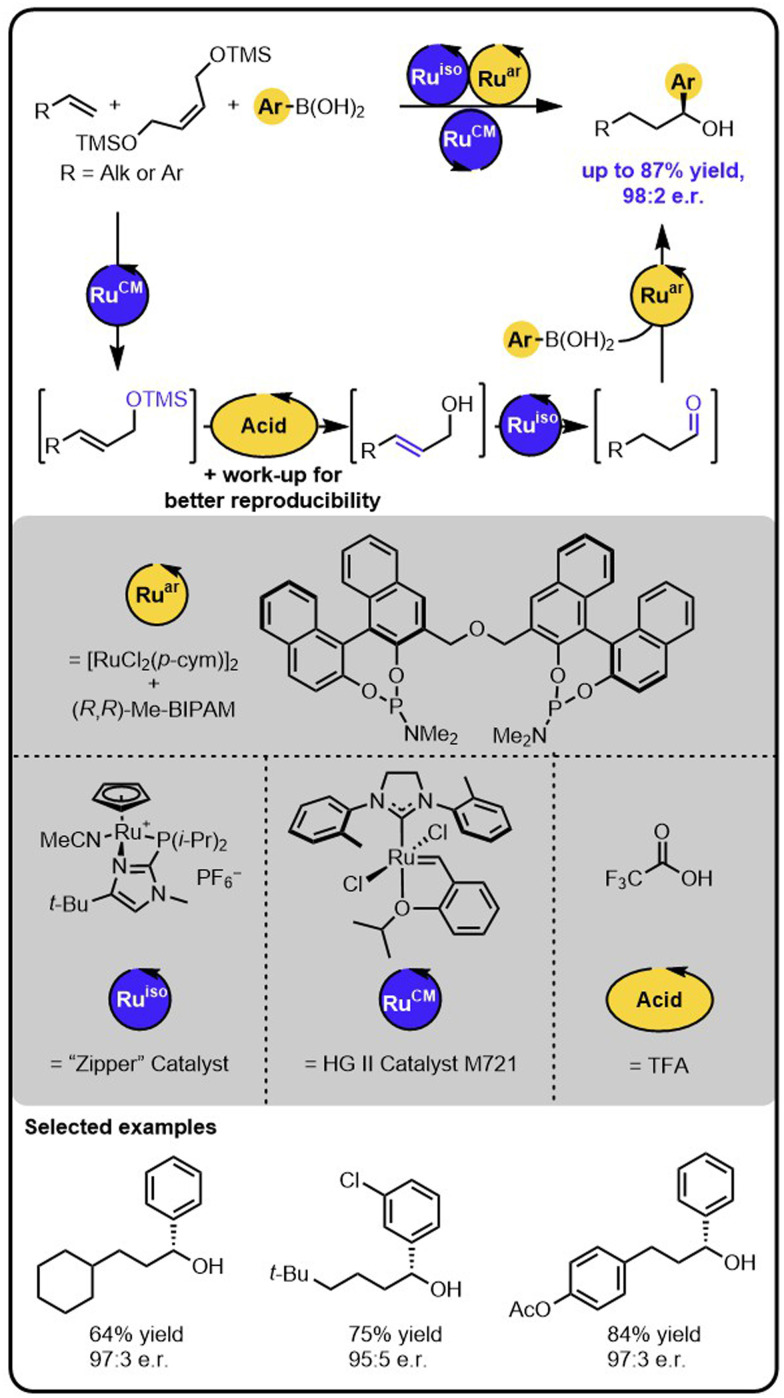
Access to enantioenriched secondary benzylic alcohols starting from alkenes, a TMS-protected diol, and boronic acids under the Ru/Acid/Ru/Ru sequential quadruple-catalytic relay.

The above-described method of synthesis of enantioenriched secondary alcohols was further expanded by incorporating a metathesis step enabling to obtain the target products starting from alkenes (Scheme 5).^44^ In this case, in the first step, an alkene undergoes olefin cross-metathesis with a protected symmetrical diol yielding a protected allylic alcohol, which then undergoes the deprotection-isomerization-hydroarylation sequence. The sequence could be conducted with a deprotected diol as well; however, the use of a TMS-protected diol resulted in the formation of the products in higher yields. The deprotection step was triggered by the addition of an acid that was subsequently removed by washing the reaction mixture with an aqueous base solution. Because the isomerization catalyst was found to be water sensitive, the removal of the aqueous phase followed by drying the reaction mixture with sodium sulphate was essential for reproducibility. Given the minimal workup, the overall transformation is formally a two-step process, rather than a single sequential-relay. However, it does provide a stepping-stone to developing relays with more than 2 catalysts. Moreover, this example of multiple catalysts working together demonstrates the potential of the approach for a rapid build-up of complexity from multiple simple starting materials.

**Scheme 6:**
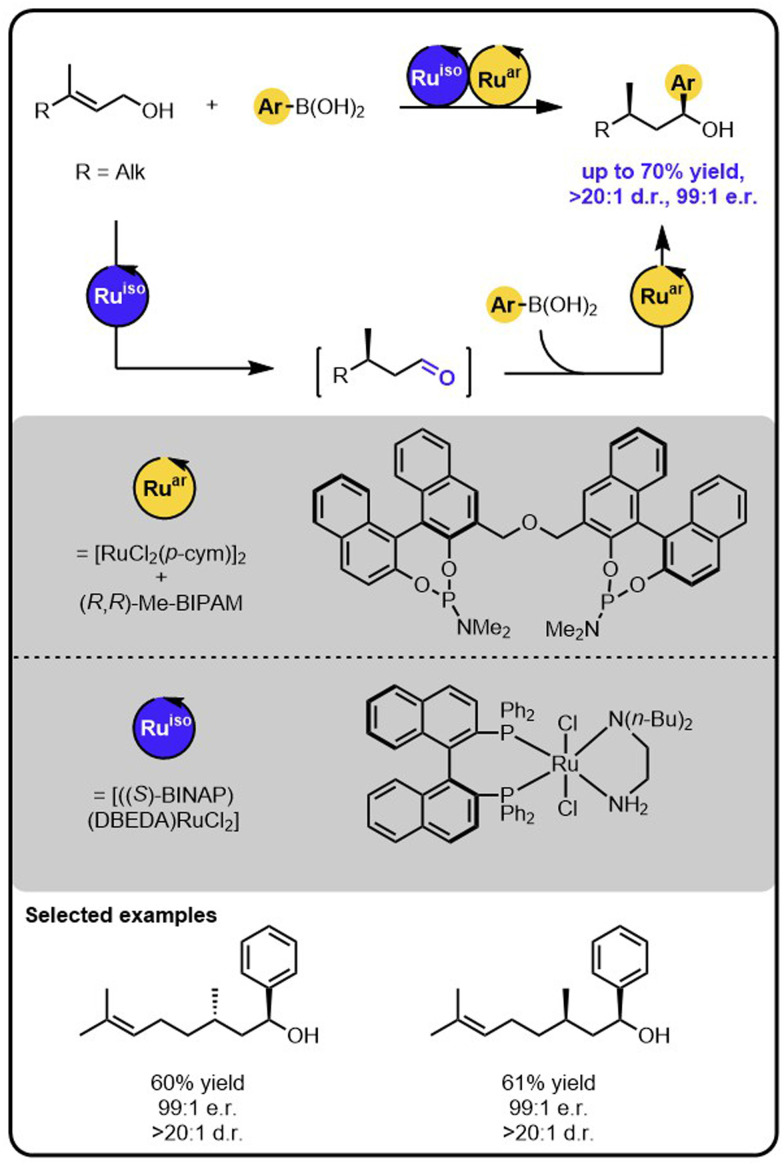
Enantioselective and diasterodivergent synthesis of secondary benzylic alcohols bearing 1,3-adjacent stereocentres from readily available allylic alcohols bearing a prochiral double bond under the Ru/Ru sequential dual-catalytic relay catalysis.

Sequential isomerization-hydroarylation relay catalysis also proved attractive for granting access to secondary benzylic alcohols bearing 1,3-adjacent stereocentres from readily available (natural) allylic alcohols bearing a prochiral double bond (Scheme 6).^44^ Because each step of the relay is executed by a different catalyst that exerts full control over one of the formed chiral centres, all four diastereoisomers of the products could be obtained selectively by varying the stereoisomers of the catalysts. In addition, the formation of one of the stereoisomers of the product could be controlled by the configuration of the double bond of the starting material.

### β-Functionalization of primary alcohols

**Scheme 7:**
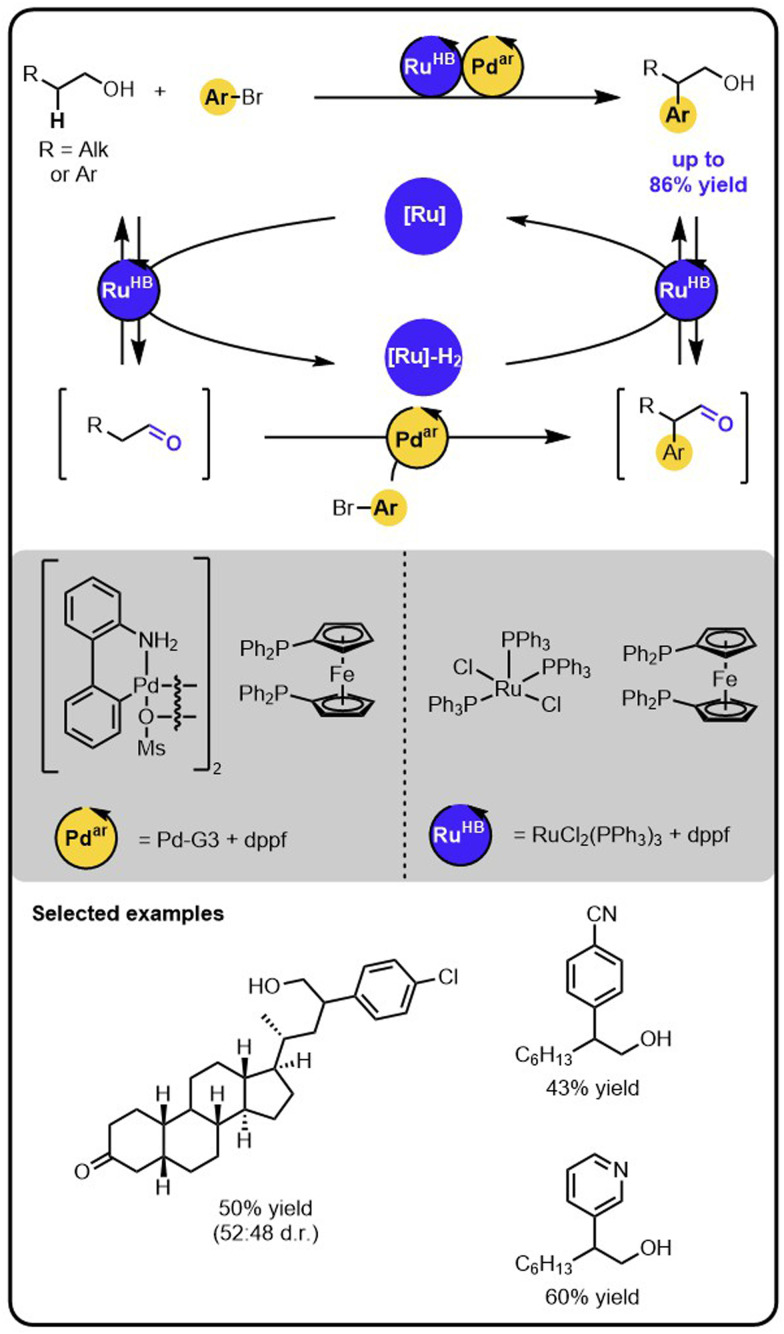
Direct β-arylation of alcohols with aryl bromides under the dual-catalytic Ru/Pd relay.

Our group reported an example of an orthogonal relay that enables the direct β-arylation of primary aliphatic alcohols with aryl bromides (Scheme 7).^45^ The strategy merges Ru-catalysed hydrogen borrowing reactivity,^26,27^ mediating reversible activation of the alcohol to a transient aldehyde intermediate, with a Pd-catalysed arylation of the (activated) aldehyde intermediate.^46–48^ Specifically, the initial oxidation of the alcohol to the aldehyde intermediate is carried out by the Ru-catalyst that temporarily stores the hydrogen. The aldehyde undergoes the arylation with aryl bromide on the Pd-catalyst. Lastly, the arylated aldehyde is reduced to the corresponding aryl alcohol by the Ru-catalyst using the borrowed and stored hydrogen. Therefore, this protocol of β-arylation of alcohols is redox-neutral, with no stoichiometric oxidants or reductants used, despite the oxidation and reduction steps involved in the relay.

The striking differences between the above-described catalytic orthogonal relay and the isolated reactions are revealed by the kinetic features. In the Ru/Pd relay, for instance, deuterium labelling studies have demonstrated that the rate-limiting step is the enolate formation from the transient aldehyde. In contrast, for the isolated arylation of the aldehyde, kinetic studies indicate that oxidative addition of the aryl bromide to the palladium catalyst becomes the slowest step. These changes in rate-limiting steps are likely a result of operating under significantly different concentration regimes. In the relay, the transient aldehyde intermediate is present at a low concentration, which is dictated by the concentration of Ru-complex and is significantly lower than the aldehyde concentration in the isolated reaction. Hence, the rate of the enolate formation becomes slow with respect to the oxidative addition of the aryl bromide in the dual-catalytic system.

The above-discussed example demonstrates that, when devising multi-catalytic systems, it is crucial to consider the kinetics and investigate the catalysts in the entire system. The simplified analysis of the isolated components of the multicatalytic transformations might lead to faulty conclusions regarding the behaviour of the complete systems.

### γ-Hydrofunctionalization of allylic alcohols

Several multicatalytic systems for selective γ-hydrofunctionalization of allylic alcohols were reported, all of which exploit the merger of the hydrogen borrowing reactivity with a range of orthogonal transformations. Common in the design is the initial oxidation of the allylic alcohol starting material to an α,β-unsaturated aldehyde or ketone intermediate with a hydrogen borrowing catalyst that temporarily stores the hydrogen. Because the oxidation step changes the reactivity of the double bond, the α,β-unsaturated carbonyl intermediate can undergo 1,4-addition with a range of nucleophiles to form a range of functionalized aldehyde intermediates, respectively. In the final stage, the latter undergo hydrogenation restoring the alcohol functionality, albeit with the double bond selectively hydrofunctionalized. Given the innate reactivity of α,β-unsaturated carbonyl compounds, the strategy does not require the use of multiple catalysts.^49–51^ However, employing multiple catalysts expands the scope of available nucleophiles and introduces precise control over the reaction selectivity, as shown by the examples discussed below.

**Scheme 8:**
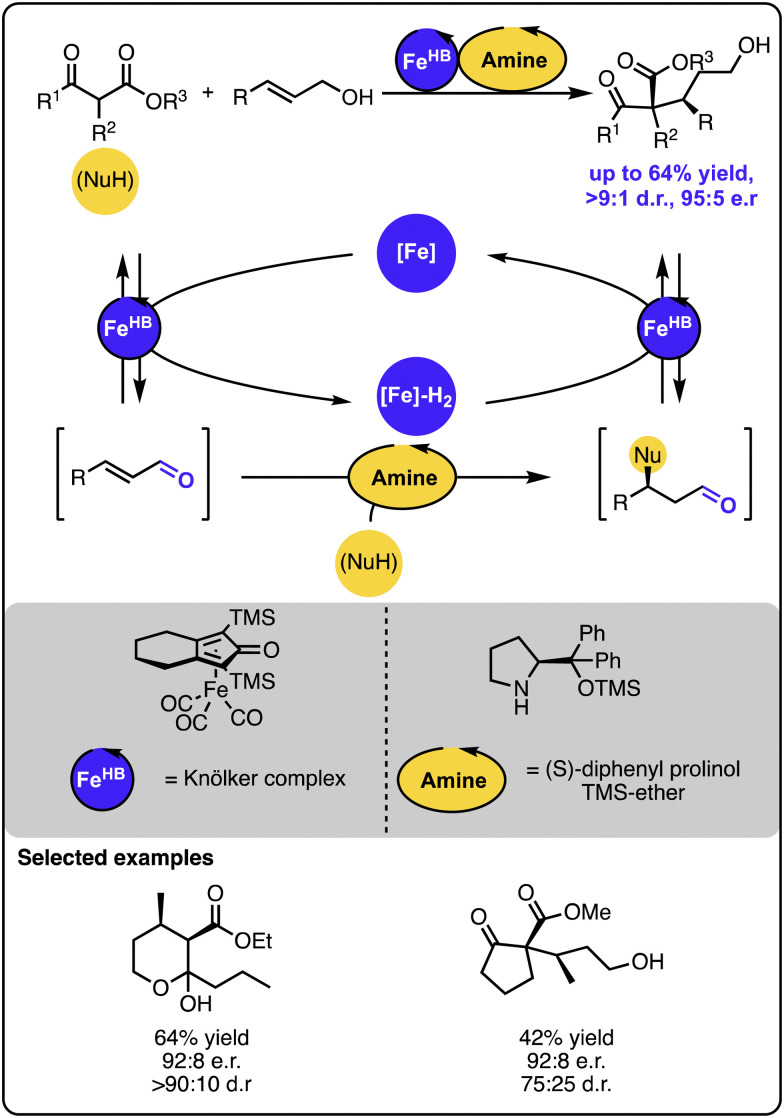
Enantioselective Michael-type addition of keto-esters to allylic alcohols under the dual iron-/organocatalytic system.

In one case, the orthogonal relay strategy was used to merge iron-based hydrogen transfer catalysis with organocatalysis to facilitate the enantioselective addition of keto-ester-based carbon nucleophiles to primary allylic alcohols (Scheme 8).^52^ Here, the 1,4-addition occurs upon the formation of an iminium intermediate between the α,β-unsaturated aldehyde intermediate and the amine organocatalyst. Given that an isolated reaction of the Michael addition of the nucleophile to the α,β-unsaturated aldehyde led to the formation of the aldehyde product with the same absolute configuration and comparable enantiomeric purity as observed in the full system, the organocatalyst seems to influence the enantio- and diastereoselectivity. The kinetic investigations indicated that the iron-based catalyst is likely involved in the rate-determining step.

In a follow-up study, the scope of the transformation was extended by incorporating catalytic copper species, which resulted in increased rates of the overall transformation.^53^ The strategy was taken even further, when the original system was combined with another reaction, enabling the direct formation of lactones from ketoesters and allylic alcohols in a one-pot procedure.^54^ This work neatly demonstrated the great ability of multicatalysis to build up rapidly complex scaffolds from relatively simple starting materials. The methodology was applied for the synthesis of the key polyketide fragment of the anticancer agent Apratoxin A.^55^ The application of the 1,4-addition sequence and the cascade shortened the overall synthesis to just 6 steps, a substantial improvement when compared with the previously reported syntheses counting between 12 to 20 steps.

The above-described work illustrates another advantage of multicatalysis. Although the Michael-type additions of related carbon nucleophiles to allylic alcohols were reported with a single catalyst that mediates the reversible oxidation,^56^ building a dual catalytic system with a catalyst for the 1,4-addition step allows for more precise control of the reaction sequence and enables controlling its enantioselectivity.

**Scheme 9:**
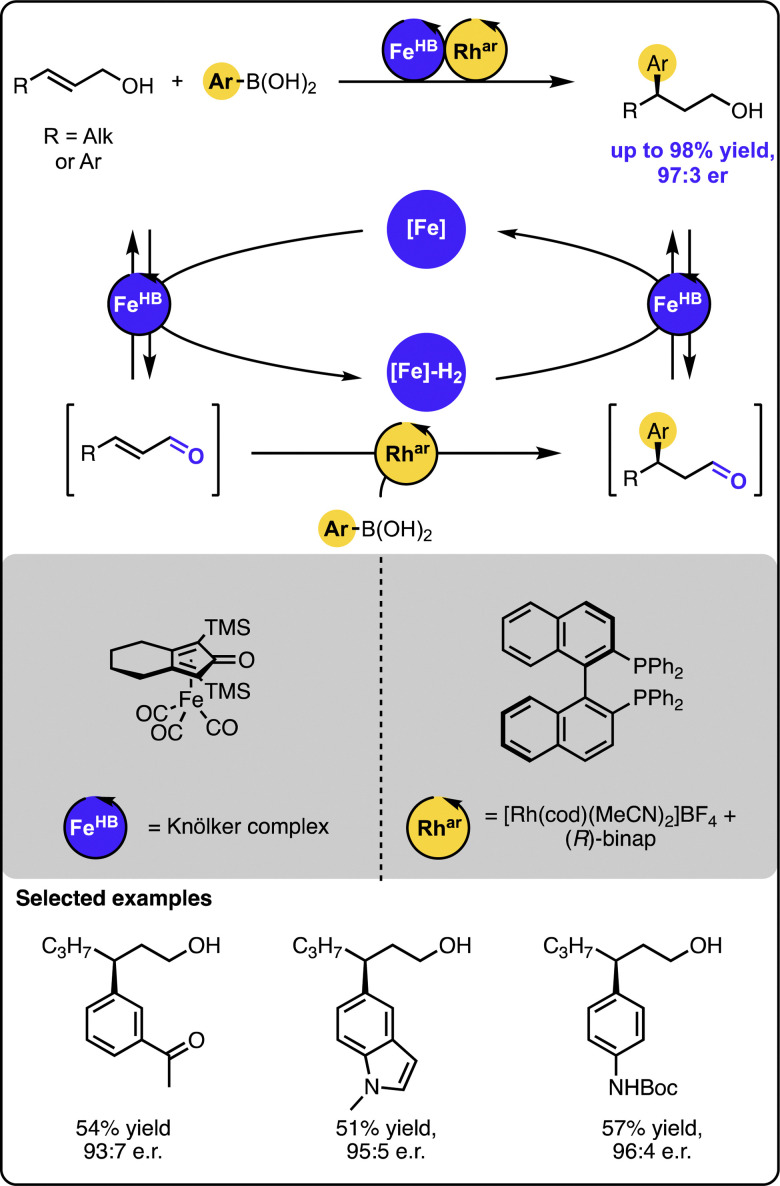
Enantioselective Michael-type addition of boronic acids to allylic alcohols under the dual-catalytic Fe/Rh relay.

In turn, our group reported the γ-hydroarylation of allylic alcohols with aryl boronic acids under dual-catalysis (Scheme 9).^45^ In contrast to the previous systems merging transition metal catalysis with organocatalysis, the reaction was driven by dual transition metal catalysis. In this case, a ruthenium or iron-based hydrogen borrowing catalyst was relayed with a rhodium-based catalyst bearing chiral (*R*)-BINAP ligand to furnish the overall transformation. The latter complex mediated the enantioselective 1,4-addition of aryl boronic acids to the transient unsaturated aldehydes. The method proved to tolerate a number of functional groups and form the target products in high yields and enantiopurity for a range of sterically and electronically-varied starting materials.

**Scheme 10:**
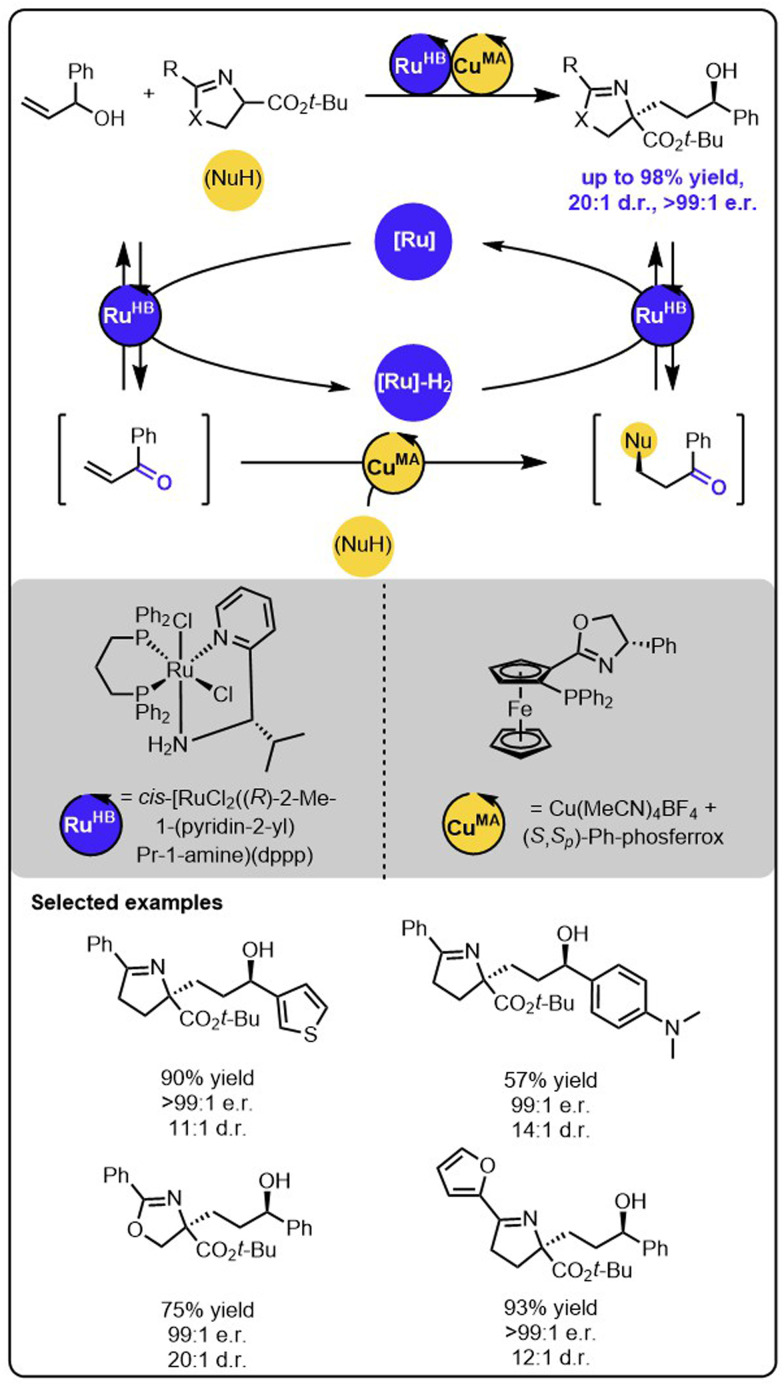
Stereodivergent synthesis of alcohols bearing 1,4-stereocentres from allylic alcohols and ketimine esters under the dual-catalytic Ru/Cu system.

While the above-described functionalizations of primary allylic alcohols occur to form the products with one stereogenic centre, the reactions with secondary allylic alcohols may lead to the formation of products with two stereogenic centres, thereby introducing the need for another level of stereoselectivity control. Wang and coworkers recently reported a ruthenium/copper relay system for the functionalization of secondary allylic alcohols to construct alcohols bearing 1,4-adjacent stereogenic centres (Scheme 10).^57^ In this case, the transiently formed α,β-unsaturated ketone undergoes the enantioselective copper-catalyzed 1,4-addition of the ketimine ester nucleophile. Then, the modified ketone is stereoselectively reduced to the alcohol through the chiral Ru complex with the hydrogen borrowed in the initial step. Importantly, the employment of two different metal catalysts allows precise control over each reaction occurring within the relay. The chirality of the newly formed stereogenic centres could be controlled by using a suitable chiral copper complex during the Michael addition and the chiral ruthenium complex during the ketone reduction. Because the configurations of both newly formed stereogenic centres are strictly controlled by the distinct chiral catalysts, all possible stereoisomers of the product could be synthesized by selecting suitable enantiomers of ruthenium and copper complexes.

### Site-selective isomerization of sugars

**Scheme 11:**
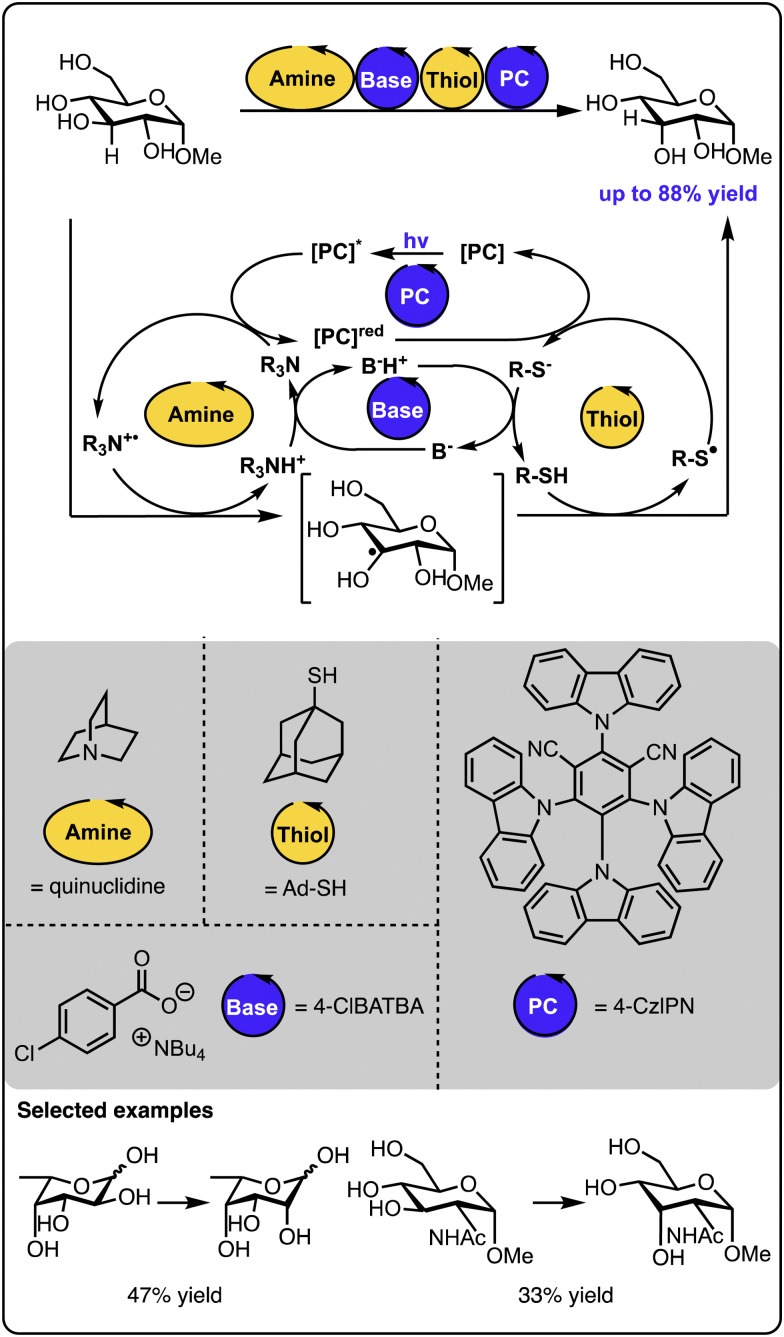
Site-selective epimerization of sugars operating under photocatalysis with two hydrogen atom transfer processes.

An excellent example of utilizing multicatalysis to access natural products, which are difficult to isolate from natural sources, was reported by the group of Wendlandt (Scheme 11).^58^ Here, rare sugar isomers were synthesized from biomass-produced carbohydrates available in large amounts. Combining two hydrogen atom transfer processes allowed for the selective altering of the stereogenic configuration at one of the carbon positions. In this instance, the catalytic quinuclidinium radical cation abstracts the hydrogen atom from the sugar starting material. The generated radical sugar intermediate then abstracts the hydrogen atom from a thiol donor, generating the product diastereoselectively (along with a thiyl radical). A base and a cyanoarene-based photocatalyst regenerate both catalytic species – the quinuclidinium radical cation and the thiol, closing all the catalytic cycles. In such processes, it is necessary to ensure that each of the catalysts involved in the hydrogen atom transfer steps are selective since any cross-reactivity could cease the target transformation. Follow-up studies revealed that sugars and other polyols could be selectively epimerized at different positions upon fine-tuning the catalysts and the reaction conditions.^59^ Similarly, photoredox catalysis and auxiliary additives allow for the epimerisation of *trans*-diols to *cis*-diols as reported by the group of MacMillan or the opposite direction – from *cis*-diols to *trans*-diols as demonstrated by the group of Wendlandt.^60,61^ Overall, these studies demonstrate that leveraging two catalysts operating under photocatalytic conditions can drive thermodynamically uphill reactions, thanks to kinetic control over the product formation and the continuous supply of energy by light.

## Conclusion

Illustrated herein with the examples of different methods enabling diversified functionalisations of aliphatic and unsaturated alcohols, multicatalysis emerges as a dynamically developing field. Attractively, many multicatalytic strategies offer the opportunity for a rapid increase in the complexity of simple starting materials by reducing the number of tedious purification steps while maintaining high yields and, where applicable, the stereoselectivity of the overall transformation. This advantage leads to improving the cost, time, and material efficiency of the transformations. In addition, multicatalytic systems offer the possibility of directly modifying poorly reactive positions of molecules, even in the presence of other innately more reactive sites. Furthermore, multicatalysis offers the possibility of designing novel transformations, which are either tedious or impossible when performed in a non-catalytic fashion or by a classical catalytic approach. Overall, developing one-pot processes can lead to remarkable breakthroughs in the synthesis of valuable chemicals and materials.^62^

Crucial to further leveraging the potential of multicatalysis as a synthetic tool is to gain deeper mechanistic insight into the created systems. To fully capitalise on the remarkable capacity of multicatalysis, it is key to develop a different mindset when designing (multi)catalytic systems. The most productive catalysts developed for simple environments, *i.e.*, isolated reactions conducted separately, can prove to be inefficient in complex settings of reaction mixtures, *e.g.*, a network of cooperative reactions. Hence breaking down the system into simpler pieces might not be the most effective way of analysing and devising a multicatalytic system. Overall, learning how catalysts behave in complex mixtures can provide the opportunity to fine-tune them and design even more potent systems.

In a broader context, multicatalysis has the potential to unlock the nature-inspired synthesis of organic molecules. Starting from simple resources and efficiently yielding complex products is a highly attractive prospect for chemists. By further designing and investigating the systems that arise when multiple catalysts are combined, the development of even more complex systems can be facilitated. This, in turn, could lead to not only more straightforward transformations but also enable high control over the outcome, all while increasing the overall efficiency of the process, the holy grail of practical catalysis.

## Conflicts of interest

There are no conflicts to declare.

## Supplementary Material
